# The NBS Scale of Spectral Irradiance

**DOI:** 10.6028/jres.093.003

**Published:** 1988-02-01

**Authors:** James H. Walker, Robert D. Saunders, John K. Jackson, Donald A. McSparron

**Affiliations:** National Bureau of Standards Gaithersburg, MD 20899

**Keywords:** blackbody, calibrations, radiometry, response linearity, slit-scattering function, spectral irradiance, standards

## Abstract

This paper describes the measurement methods and the instrumentation used in the realization and transfer of the NBS scale of spectral irradiance. The basic measurement equation for the irradiance realization is derived. The spectral responsivity function, linearity of response, and “size of source” effect of the spectroradiometer are described. The analysis of sources of error and the estimates of uncertainty are described. The assigned uncertainties (3*σ* level) in spectral irradiance range from 2.2% at 250 nm to 1.0% at 654.6 nm to 6.5% at 2400 nm.

## 1. Introduction

Spectral irradiance, denoted *E*_λ_, is defined as the radiant flux of wavelength λ incident on a surface per unit wavelength interval and per unit area on the surface. Mathematically

$$E_{\lambda }=d^{2}\Phi /\text{d}\lambda \cdot dA,$$ (1)

where d^2^Φ is the element of incident flux and dλ and d*A* are the elements of wavelength and area respectively.

The National Bureau of Standards (NBS) presently issues two types of spectral irradiance standards. Type FEL (ANSI designation) lamps, modified to a medium bipost base, are calibrated as standards of spectral irradiance at 31 wavelengths over the spectral range 250 to 2400 nm. Deuterium lamp standards of spectral irradiance are calibrated at 16 wavelengths over the spectral range 200 to 350 nm and at a lower accuracy than the type FEL lamps. Both these lamp standards are designated in NBS Special Publication 250 [1].

In 1963, NBS established a scale of spectral irradiance [2]. In the early 1970’s an improved scale was developed [3] with uncertainties about 1/3 those of the earlier scale. The detailed techniques for realizing this scale have undergone several evolutionary changes in the past decade. This paper is a description of the current process of realization of the NBS spectral irradiance scale and of the current procedures for the routine spectral irradiance calibrations.

Modified type FEL lamps are routinely calibrated from 250 to 2400 nm. Deuterium lamps are routinely calibrated from 200 to 350 nm. The spectral irradiance values transferred to the deuterium lamps in the spectral range 200 to 250 nm are based on the hydrogen and blackbody line arcs developed primarily for use in the vacuum ultraviolet [4]. From 250 to 350 nm the reported spectral irradiance values are transferred from the modified type FEL lamps. The equipment used for the deuterium lamp calibrations is identical to that used for the modified type FEL lamp calibrations, and the measurement procedures are very similar. The body of this paper will be limited to a description of the calibration of the modified type FEL lamps. Detailed information on the calibration services appears in a separate document [5].

## 2. Scale Derivation and Transfer

The NBS scale of spectral irradiance is derived from the NBS scale of spectral radiance [6] which is based on a realization of the International Practical Temperature Scale (IPTS-68) starting with a gold-point blackbody [7]. The average spectral radiance over the exit aperture of a special integrating sphere source is determined and then the flux from the sphere source which enters the receiving aperture of the spectroradiometer is calculated. This technique is used to determine the spectral irradiance at the detector receiving aperture and thus establishes a spectral irradiance scale. As a matter of convenience the scale is transferred to a group of four, 1000 W, quartz-halogen lamp primary working standards using an averaging sphere-monochromator combination designed for spectral irradiance measurements. These lamps are used to maintain the NBS scale of spectral irradiance. The lamps are recalibrated every 50 to 100 burning hours.

**Figure 1:**
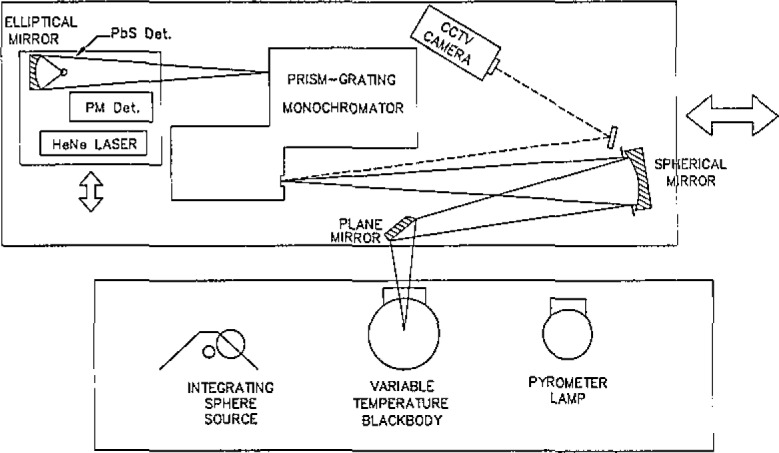
Spectral radiance measurement setup.

**Figure 2:**
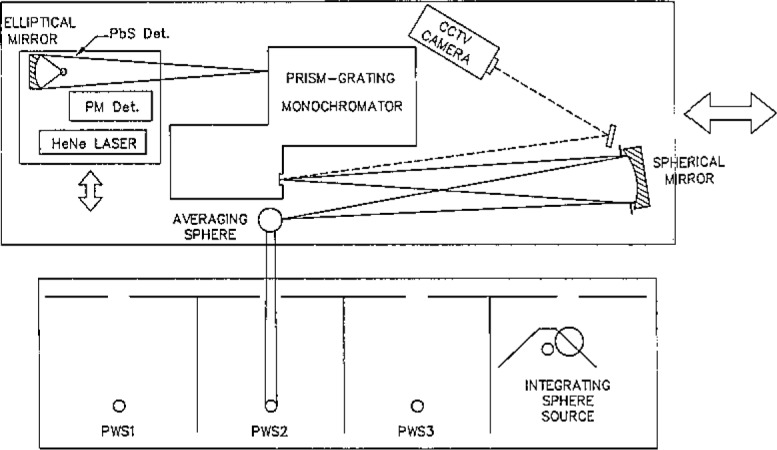
Spectral irradiance measurement setup.

Figure 1 shows the setup used to measure the spectral radiance of the special integrating sphere source. Figure 2 shows the setup used to transfer the spectral irradiance scale to a group of primary working standards.

**Figure 3:**
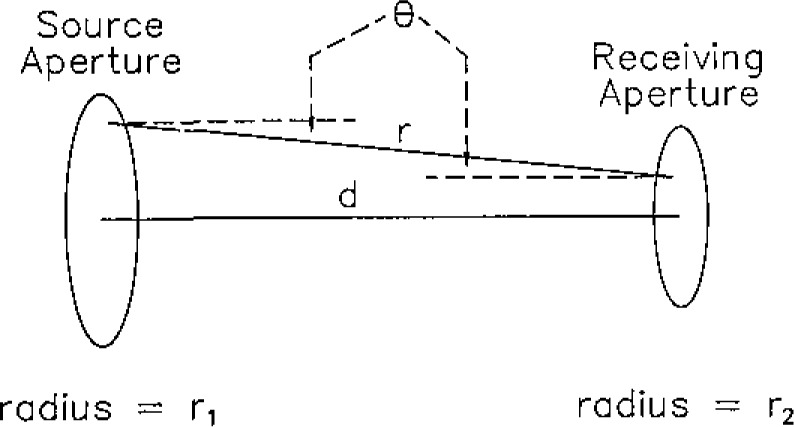
Irradiance calculation geometry.

The geometry used for the spectral irradiance determination is shown in figure 3. The following method is used to determine the spectral irradiance at the receiving aperture of the spectroradiometer produced by the integrating sphere source. The spectral irradiance, *E*_λ_, at the receiving aperture due to the spectral radiance, *L*_λ_, at any point on the source aperture is

$$E_{\lambda }=\int_{\omega }L_{\lambda }\cdot d\omega ,$$ (2)

where *ω* is the solid angle defined by the receiving aperture and a point on the source aperture. To calculate the flux at the receiving aperture due to the entire source aperture, it is necessary to integrate over the entire projected area of the source aperture

$$\Phi_{\lambda }=\int_{A}\int_{\omega }L_{\lambda }\cdot d\omega \cdot dA,$$ (3)

where d*A* = d*x*·d*y*·cos*θ*. *L*_λ_ is a function of *θ,ϕ*, *x*, and *y* so that

$$\Phi_{\lambda }=\int_{A}\int_{\omega }L_{\lambda }(\theta ,\phi ,x,y)\cdot \cos\theta \cdot d\omega \cdot \text{dx}\cdot \text{dy},$$ (4)

where:

*θ* is the angle between the normal to the surfaces of the apertures and a line connecting a single point on each aperture,
*ϕ* is the azimuthal angle,
*x* is the horizontal location of a point on the source aperture,
*y* is the vertical location of a point on the source aperture.

Assuming that the source is Lambertian and nearly uniform, *L*_λ_(*θ*, *ϕ*, *x, y*) can be replaced by an average radiance *L*_λ_ to give

$$\Phi_{\lambda }={\bar{L}}_{\lambda }\cdot \int_{A}\int_{\omega }\cos\theta \cdot d\omega \cdot dx\cdot dy,$$ (5)

where:

d*ω* = cos*θ*/*r*^2^·d*x*′d*y*′,
*x*′,*y*′ is a point on the receiving aperture,
*r* is the slant distance from *x*′, *y*′ to *x*, *y.*

This gives

$$\Phi_{\lambda }={\bar{L}}_{\lambda }\cdot \int_{A_{\text{SA}}}\int_{A_{\text{RA}}}\cos^{2}\theta /r^{2}\cdot dx^{\prime }\cdot dy^{\prime }\cdot dx\cdot dy,$$ (6)

where:

*A*_SA_ is the area of the source aperture,
*A*_RA_ is the area of the receiving aperture.

For circular, coaxial source and receiving aptures, this integral evaluates to

$$\Phi_{\lambda }={\bar{L}}_{\lambda }\cdot \pi^{2}/2\cdot [R^{2}-{\left(R^{4}-4\cdot r_{1}^{2}\cdot r_{2}^{2}\right)}^{1/2}],$$ (7)

where:

$R^{2}=d^{2}+{r_{1}}^{2}+{r_{2}}^{2}$,
*d* is the normal distance between source and receiving apertures,
*r*_1_ is the radius of the source aperture,
*r*_2_ is the radius of the receiving aperture.

A more convenient expression is

$$\Phi_{\lambda }={\bar{L}}_{\lambda }\cdot (\pi \cdot r_{1}^{2})\cdot (\pi \cdot r_{2}^{2})/R^{2}\cdot [1+\delta +2\cdot \delta^{2}+5\cdot \delta^{3}+\ldots ],$$ (8)

where $\delta =(r_{1}^{2}\cdot r_{2}^{2})/R^{4}$. Finally,

$$E_{\lambda }=\Phi_{\lambda }/A_{\text{RA}}={\bar{L}}_{\lambda }\cdot (\pi \cdot r_{1}^{2})/R^{2}\cdot [1+\delta +\ldots ].$$ (8a)

The final step is to compare the spectroradiometer outputs produced by the integrating sphere source and each working standard.

Once the primary working standards have been calibrated, they are used to measure the spectral irradiance of test lamps. Modified type FEL test lamps are calibrated in groups of twelve.

## 3. Measurement Apparatus

Spectral radiance and spectral irradiance calibrations are performed on the NBS Facility for Automated Spectroradiometric Calibrations (FASCAL) [8]. Block diagrams of the measurement apparatus are shown in figures 1 and 2. The principal components are:

1. Variable-Temperature Blackbody
2. Sources
  Pyrometer Lamp
  Special Integrating Sphere Source
  Spectral Irradiance Primary Working Standards
  Test Lamps
3. Spectroradiometer
  Fore-optics
    Avenging Sphere
    Mirrors and Entrance Slit Masks
  Monochromator
  Detectors
4. Control and Data Acquisition System

### 3.1 Variable-Temperature Blackbody

The variable-temperature blackbody is used over a temperature range from about 800 °C to about 2400 °C.

**Figure 4:**
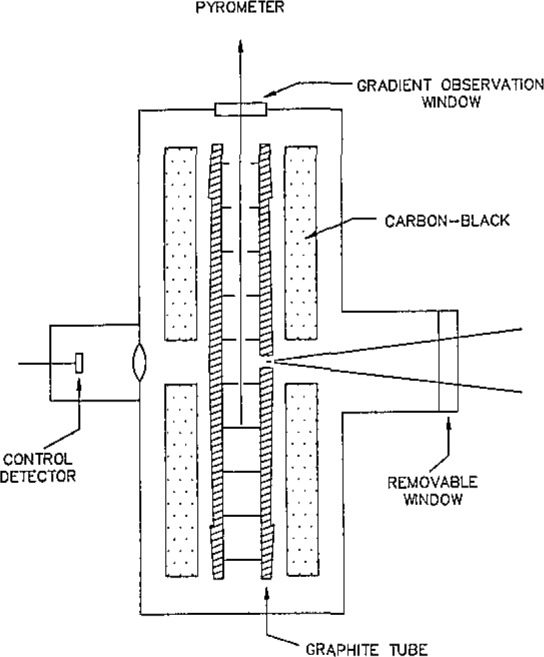
Variable-temperature blackbody schematic.

A schematic cross section of the variable-temperature blackbody is shown in figure 4. The blackbody cavity is located in the central portion of a high density graphite tube, which is resistively heated in an argon atmosphere. Electric current is supplied to the graphite tube through water-cooled electrical connections at each end of the tube. The tube is surrounded by a double-walled graphite radiation shield, with carbon black fill between the walls. This assembly is surrounded by a water-cooled metal housing, with an observation port which can be sealed during evacuation of the atmosphere within the housing prior to flushing with argon. A window is provided at the top of the housing for visual pyrometer observation of the temperatures along the tube interior. A second window at the rear of the housing allows radiation from the rear wall of the graphite tube to fall on a silicon photodiode. The photodiode provides a signal for automatic control of the saturable-reactor power supply for the tube. A germanium photodiode, whose response extends further into the infrared region, replaces the silicon cell for operation at temperatures below 1000 °C. The blackbody mounting provides adjustment in two angular and three translational degrees of freedom, allowing for precise positioning and radiometric scanning over the target area and the beam solid angle.

**Figure 5:**
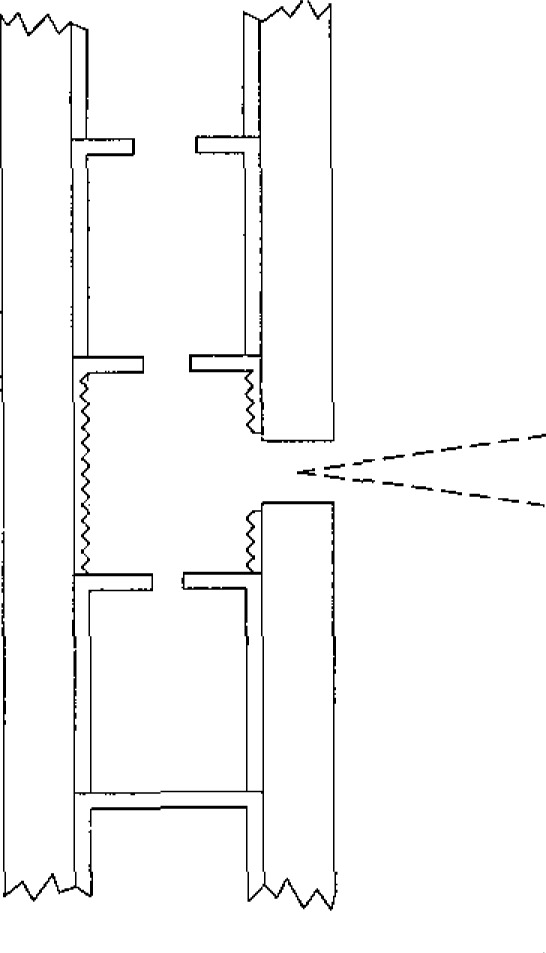
Central section of variable-temperature blackbody.

The graphite tube is about 200 mm long, with an inner diameter of about 11 mm. The outer surface is tapered to improve temperature uniformity along its length. The wall is about 4 mm thick at mid-length where a 2 mm diameter hole in the wall allows for observation of the emitted flux. The tube is partitioned into small cylindrical sections by a series of thin graphite disks separated by thin graphite cylinders located at intervals along the bore. Holes in the graphite disks permit measurement of the temperatures in the middle and upper sections with a visual pyrometer. The holes vary in diameter from 6 mm for the uppermost disk to 0.75 mm for the disk below the central section. The central cylindrical section, which provides the observed flux, is 9 mm high and 10 mm in diameter. The inside wall is threaded to reduce its partial reflectivity [9,10]. Figure 5 shows a cross-sectional view of the central section.

The blackbody emissivity has been assessed by measurements of the solid angle subtended by the cavity opening, the partial reflectivity of the graphite material [9], the temperature gradients, and the absorption by gases [10]. The solid angle subtended at the rear wall of the cavity by the inner edge of the observation hole is about 0.03 sr. The measured partial reflectivity of the graphite is 0.02 sr^−1^. The measured temperature gradient over the length of the viewing cavity is less than 1 K. Experimental investigations of possible absorption of radiation by gases has disclosed only weak absorption lines at 589 and 589.6 nm (Na) and at 766.5 nm (K). The resulting estimate of emissivity is 0.9990±0.0005.

### 3.2 Sources

#### 3.2.1 Pyrometer Lamp

This lamp is used as a secondary standard for realizing the spectral radiance scale. It is a highly stable vacuum tungsten-strip lamp which is operated at a single current to produce a spectral radiance of about eight times that of a gold-point blackbody at 654.6 nm (about 1530 K radiance temperature). The lamp drift rate is less than 0.02% per 100 hours when operated at a single current level.

#### 3.2.2 Special Integrating Sphere Source

This source has been specially constructed to be unpolarized and to have high output in the IR part of its spectrum. It consists of a heat-sinked, water-cooled integrating sphere with a 1000 W quartz-halogen, modified type FEL lamp mounted next to the entrance port. The integrating sphere is 5.0 cm in diameter with a 23 mm diameter entrance port and a 20 mm diameter exit port located about 100° from the entrance port. The inside sphere wall is coated with pressed high purity polytetrafluoroethylene (PTFE) [18] to give high reflectivity in the IR. A modified type FEL lamp is mounted with its envelope about 3 mm from the entrance port of the sphere and located so that it does not directly irradiate the inside wall of the sphere opposite the exit port. The sphere itself is made of copper and is mounted in a heat-sinking copper plate. Copper tubing is soldered to the sphere and plate and the entire assembly is water-cooled to prevent the source from overheating. A precision circular aperture whose area has been accurately measured is attached at the exit port.

**Figure 6:**
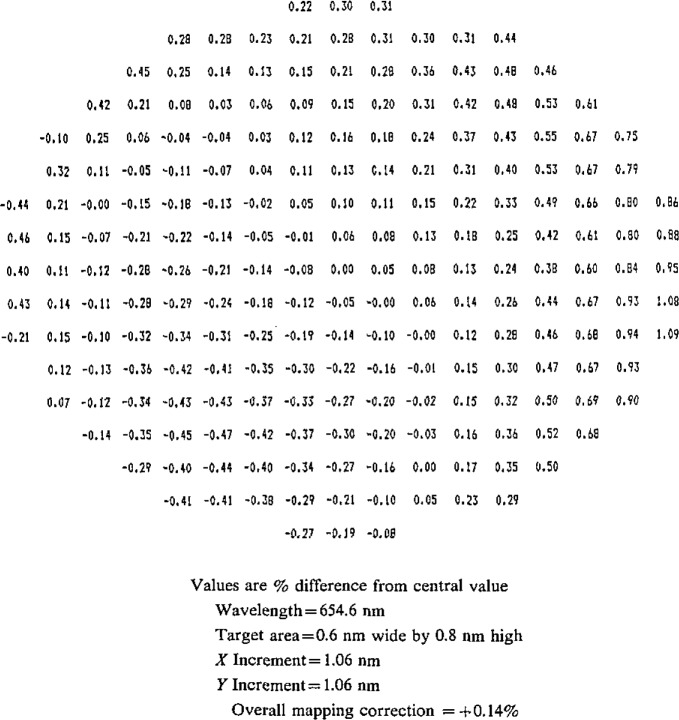
Mapping profile of integrating sphere aperture.

Because of the multiple reflections in the integrating sphere, entering radiation is randomized, producing a uniform, depolarized radiant flux at the exit port. The uniformity is verified when the exit port aperture is mapped during the irradiance realization procedure (fig. 6 shows a typical mapping profile). Depolarization was tested at 650 nm using an unpolarized source and a linear polarizer and found to be complete within the measurement precision of 0.1% (3*σ* level).

#### 3.2.3 Spectral Irradiance Primary Working Standards

Four 1000 W quartz-halogen, modified type FEL lamps were selected as primary working standards. This type lamp has a clear bulb and a tungsten coiled-coil filament (CC-8) and has a rated life of 500 hours at 120 V. Before calibration, the lamp base is converted to a medium bipost base and the base structure is encapsulated in an epoxy-ceramic compound. The posts that form the bipost base are 6.35 mm (1/4 in) diameter cylindrical stainless steel rods that extend 20.64 mm (13/16 in) from the bottom of the epoxy-ceramic block. The posts are spaced 22.23 mm (7/8 in) between centers. A metal plate bearing the lamp identification number and indicating the electrical polarity is attached to the rear surface (side away from the spectroradiometer) of the epoxy-ceramic block.

#### 3.2.4 Test Lamps

The test lamps are also modified type FEL lamps. A lamp screening process is used to select test lamps suitable for calibration. Lamps are annealed and then are checked for stability, emission lines or absorption bands, and for variations in goniometric output.

### 3.3 Spectroradiometer

#### 3.3.1 Fore-optics

##### Averaging Sphere

The averaging sphere is 2.5 cm in diameter with a 1 cm^2^ area precision circular entrance port and a 3×12 mm exit port located about 100° from the entrance port. The inside sphere wall is coated with pressed high purity PTFE. This material has been found to fluoresce at certain wavelengths under certain conditions [19], but when the sources being compared have approximately the same spectral distribution, fluorescence is not a problem. The radiation entering the sphere is randomized by multiple reflections in the sphere, thus producing uniform, depolarized radiant flux at the exit port. This uniformity was verified to within the measurement precision of 0.1% (3*σ* level) by radiometrically scanning the exit port of the sphere. Depolarization was tested at 500 nm and 650 nm using an unpolarized source and a linear polarizer and found to be complete within the measurement precision of 0.1% (3*σ* level).

The difference in the solid angle of irradiation for the irradiance lamp and the integrating sphere source is only a problem when the reflectance of the averaging sphere wall is not uniform. This high purity, 3 mm thick PTFE sphere coating provides this uniformity, and it was verified by determining the spectral irradiance of a lamp mirror-system [3] whose solid angle could be varied. Negligible difference (<0.1%) in the results was observed over the range of solid angles viewed (the conical full angle was varied from 1.85° to 10°).

##### Mirrors and Entrance Slit Masks

In the radiance measurement mode, the radiance source is imaged with unit magnification onto a polished stainless steel mask placed directly in front of the entrance slit of the monochromator. In the irradiance measurement mode, the exit port of the averaging sphere is imaged with unit magnification onto a different polished stainless steel mask. The mask determines the height of the system field stop (source target area) and the entrance slit determines the width. In the radiance mode the stop dimensions are 0.6 mm wide by 0.8 mm high. Also, in this mode the optic axis lies within 1.5° of the axis of the spherical mirror to minimize aberration [11]. In the irradiance mode the stop dimensions are approximately 2 mm wide by 10 mm high. In this mode the off-axis angle is slightly larger, but in this case the image quality is less important because of the homogeneity of the flux exiting the averaging sphere.

#### 3.3.2 Monchromator

A prism-grating double monochromator is employed to minimize spectral scattering and to avoid multiple orders. It is used over the wavelength range of 200 to 2400 nm. The dispersion varies with wavelength from about 1 to 4 nm/mm. The entrance aperture (solid angle) is rectangular in shape, with a vertical angle of 7° and a horizontal angle of 3.5°. The wavelength setting is calibrated against Hg line standards from 250 to 580 nm and against Th line standards from 800 to 2400 nm. (See reference [5] for specific lines.) The wavelength calibration is repeatable to within 0.05 nm. The entrance, intermediate, and exit slits are adjustable together as a unit from 0.01 to 3.0 mm, resulting in a nearly triangular-shaped spectral bandpass.

#### 3.3.3 Detectors

Two interchangeable detectors are used to cover the wavelength range of the spectroradiometer. For the 200 to 850 nm range, an end-on 11-stage photomultiplier with quartz window and S-20 spectral response is placed behind the exit slit. The detector is cooled to 258 K with a thermoelectric cooler. The anode current is amplified and converted to a 0 to 10 V signal by a programmable dc amplifier. To ensure linearity of response, the high voltage applied to the detector is normally selected to restrict the detector current to 500 nA or less.

A lead sulfide detector, cooled to 240 K by a thermoelectric cooler, is used for the 800 to 2400 nm range. The detector and the exit slit are placed at the foci of an ellipsoidal mirror, which images the exit slit upon the detector with a demagnification of about 7. The detector output is amplified and converted to a 0 to 1 V signal by a phase-sensitive lock-in voltmeter, which is keyed to a 78 Hz sector disk placed just before the plane mirror in the radiance mode or just after the exit port of the averaging sphere in the irradiance mode.

The signal from either detector-amplifier combination is fed to a 5 1/2 digit voltmeter, capable of integration times ranging from one second to several minutes. To facilitate alignment of optics or sources, a HeNe laser is placed at the detector position, so that its beam passes through the monochromator and fore-optics in the reverse direction.

### 3.4 Control and Data Acquisition System

After initial alignment, the FASCAL system permits control of the entire measurement process from a remote operator console. Component positions, instrument settings, sequence of operations, and data collection are effected by either stored computer programs, operator commands, or a combination of the two.

The system is directed by a microcomputer equipped with a CRT terminal and keyboard and a high-speed disk system for program and data storage. A modular interface controller [12] provides the link between instruments and computer. All measurement signals are multiplexed into the digital voltmeter through the interface scanner, and the instruments are remotely programmed and controlled through interface modules. All instrument settings and signal outputs are printed and stored on disk for later analysis.

The spectroradiometer (fore-optics, monochromator, and detectors), a closed-circuit TV camera, and a photoelectric pyrometer are mounted on a carriage. The carriage can be moved by remote command along a linear track, to position the spectroradiometer in front of any of the sources mounted at fixed stations along the track. The average move time between stations is a few seconds, and positions are repeatable to about 0.1 mm. The TV camera presents a highly magnified image of the monochromator entrance slit mask to video displays at the spectroradiometer and at the operator console for initial source alignment and subsequent monitoring. The pyrometer is used for the initial setting of the variable-temperature blackbody to its approximate temperature.

## 4. Measurement of Instrument and Source Parameters

### 4.1 Spectral Responsivity Function

The relative spectral responsivity function of the spectroradiometer is determined by an indirect method [13]. In this method, the relative responsivity function is treated as the product of two terms, the responsivity factor and the slit-scattering function, where the responsivity factor depends only upon the wavelength of the observed flux and the slit-scattering function depends only upon the difference between the wavelength setting of the monochromator and the wavelength of the flux. This factorization of the spectral responsivity function is valid if the instrument dispersion, aberrations, scattering, and diffraction are constant over the wavelength region of interest. This assumption is valid in the central portion of the relative responsivity function, but values for the distant wings are subject to error due primarily to changes in scattering and dispersion.

The responsivity factor is obtained by spectrally scanning a continuous source standard of spectral radiance using narrow (0. 1 mm) slits. To determine the slit-scattering function, an integrating sphere irradiated by a high-powered laser is spectrally scanned by the spectroradiometer, with the slit widths set at the 0.6 mm width used in the scale realization and transfer. The plot of the output signal versus wavelength is the mirror image of the plot of the slit-scattering function versus wavelength. For a 647 nm Kr laser, the function is nearly triangular in shape with a width at half-height of 2.5 nm. Relative to the peak value, the measured values decrease to about 10^−3^ at 3 nm, 10^−4^ at 15 nm, and 10^−7^ at 70 mm from the central wavelength. At 150 nm from the central wavelength, the value decreases to 10^−8^ in the short-wavelength wing and to 10^−9^ in the long-wavelength wing. Scans with 488 nm (Ar), 514 nm (Ar), and 676 nm (Kr) yield similar results. These values were confirmed over the central and near wing portions of the function by measurements with the direct method, using a dye laser tuned through a series of wavelengths with the spectroradiometer set at a fixed wavelength [14].

The measurement at 647 nm yielded the split-scattering function used for 654.6 nm, where the spectral distribution mismatch of a variable-temperature blackbody and a gold-point blackbody requires an accurate determination of the relative responsivity function. However, the measurements in the visible cannot be applied with confidence to the short-wavelength region, since the dispersion varies by about a factor of 2.5. For this region, the central portion and near wings of the slit-scattering function are determined by scans of a spectral line discharge source, and values in the distant long-wavelength wing are deduced from a measurement of the integrated spectrally-scattered radiation. With the wavelength set at a selected value in the 200 to 250 nm region, the signal from a calibrated lamp (radiance temperature 2475 K at 654.6 nm) is recorded. A glass filter which blocks all radiation in the vicinity of the wavelength setting and passes about 90% of the radiation at longer wavelengths is inserted into the beam. The ratio of signals with and without filter is taken as the fractional contribution of spectrally scattered radiation to the signal. A second (identical) filter is added to insure that only scattered light is being observed in the filtered beam. Results with filters of different cutoff wavelengths (Corning filters CS 0-56 and CS 0-52) both indicate an integrated scattered light contribution of less than 0.2% at 225 nm. The slit scattering function calculated from this result and the known source distributions and responsivity factor are less than 10^−9^ at wavelengths greater than 200 nm from the central wavelength, in good agreement with the values measured in the visible.

### 4.2 Linearity of Response

The degree of linearity of the spectroradiometer response is determined with an automated beam conjoiner [15,16]. A beam from a constant source is split into two branches whose fluxes are independently attenuated or blocked before recombination and further attenuation. The flux from both branches measured together should equal the sum of the fluxes from each branch when measured separately (additivity). The device provides 96 levels of flux ranging over a factor of about 500. The levels are presented in random order to avoid systematic errors and are interspersed with 29 zero flux levels. A microcomputer controls the attenuating filters and records the filter positions and radiometer signals. The data is least-squares fitted to a polynomial response function to determine a correction factor by which the radiometer output signal must be multiplied to obtain a quantity proportional to radiant flux.

The response function of the spectroradiometer is dependent upon the detector-amplifier employed. With the photomultiplier tube in place (spectral range 200 to 850 nm), the instrument response at all wavelengths is linear to within 0.2% for a range of anode currents from 1 to 500 nA. Linearity measurements were performed at 900, 600, 300, and 250 nm. For currents much less than 1 nA, the signal is limited by noise. For currents greater than 1 nA the correction increases rapidly, rising to 3% at 7 μA. The anode current is restricted to less than 500 nA during measurements by selection of appropriate photomultiplier tube voltage. Correction factors for the amplifier ranges are determined from the measurement of a known electrical current and combined with the linearity correction factor.

Linearity tests of two PbS detectors resulted in a correction factor which is a linear function of the signal over the range 1 to 280 mV. The correction varies from 0.1% at 3 mV to about 9% at 300 mV. To avoid relying on large corrections, sources are typically operated at near equality in the PbS spectral region.

### 4.3 Size of Source

The “size of source” effect (signal contribution due to flux which originates outside the target area and is scattered into the measured beam by the fore-optics) is determined by observing the change in signal from a 0.6 by 0.8 mm area of a uniform diffuse source while placing various size masks on the diffuse source. The masks expose source areas which closely approximate the radiant areas of the lamp, the blackbody and the integrating sphere source used in the scale realization. As a check, the effect is also evaluated by observing changes in the near-zero signal from a “black hole” (an absorbing cavity slightly larger than the 0.6 by 0.8 mm field stop) as the various surrounding area masks are positioned. The observed differences are used to apply a correction to the signals observed in source comparisons. The effect is measured at wavelengths of 654.6 and 350 nm, and values for other wavelengths are estimated from the assumption of an inverse wavelength dependence. The correction varies from 0.04% to 0.1% at 654.6 nm depending upon the elapsed time since the last mirror recoating.

### 4.4 Polarization

The polarization properties of the spectroradiometer and the sources do not play a significant role in the spectral irradiance realization and will not be discussed here. A discussion of polarization properties can be found in reference [6].

## 5. Process of Spectral Irradiance Realization

The spectral radiance of the special integrating sphere source is determined so that it can be used as a transfer standard for determining spectral irradiance. The spectral radiance output from the center point of the integrating sphere aperture is compared to the spectral radiance output from a variable-temperature blackbody. The temperature of the blackbody is determined by comparing it at 654.6 nm to a high stability vacuum pyrometer lamp calibrated for a single temperature (about 1530 K). The spectral radiance of the integrating sphere source is determined at 31 different wavelengths from 250 to 2400 nm. The aperture of the integrating sphere is mapped at 2000, 1050, 654.6 and 300 nm and its average spectral radiance is computed for each wavelength. Figure 6 shows a typical mapping profile of the integrating sphere aperture. The mapping correction varied less than 0.1% over the range of wavelengths measured.

The spectroradiometer is changed from the spectral radiance mode to the spectral irradiance mode (see figs. 1 and 2) and the spectral irradiances from the NBS primary working standards (PWS) are compared to the spectral irradiance from the integrating sphere source (ISS). Appropriate partitions and baffles are erected to reduce scattered light to less than 0.1%. The comparisons are done at the same 31 wavelengths at which the integrating sphere source was calibrated for spectral radiance. Two separate determinations are performed on each primary working standard. The spectral irradiance of a primary working standard is determined using the relationship

$$E_{\lambda }(\text{PWS})=L_{\lambda }\cdot (\pi \cdot r_{1}^{2})/R^{2}\cdot S_{\text{PWS}}/S_{\text{ISS}},$$ (9)

where S_PWS_/S_ISS_ is the ratio of the irradiance signal from the primary working standard to the irradiance signal from the integrating sphere source. The first part of the expression comes from eq (8a) where *δ* ≈ 2·10^−16^.

The absolute output from the integrating sphere source is monitored at six wavelengths (2000, 1600, 1050, 800, 600, and 400 nm) during the 30 to 40 operating hours necessary to calibrate the primary working standards. Finally, the blackbody is used again to perform an abbreviated spectral radiance calibration of the integrating sphere source. Spectral radiance drift corrections, linear with time, for the integrating sphere source can then be made if necessary.

The measurement of the spectral radiance or spectral irradiance at a single wavelength takes from about 4 to 8 minutes, so it is only necessary for our detectors to have good short term stability.

## 6. Process of Spectral Irradiance Transfer

The four modified FEL primary working standards are used to perform spectral irradiance calibrations on test lamps. For a selected group of 12 test lamps, each lamp is measured four times, once in each of the four source positions and once against each of the four primary working standards. The screening and selection of test lamps can take several weeks and the calibration procedure for 12 test lamps takes from 2 to 3 weeks. Details of the routine spectral irradiance calibrations appears in a separate document [5].

## 7. Scale Realization Data Analysis

The spectral irradiance scale is generally realized at the following 31 wavelengths:
250 nm  600 nm260  654.6270  700280  800290  90030010503101150320120033013003401540350160040017004502000500210055523002400Certain wavelength regions in the IR are skipped (around 1400 nm and 1800 to 1980 nm) in order to avoid atmospheric absorption bands.

Since the total operating time for each primary working standard during a complete scale realization is relatively short (8 to 12 hours), no effort is made to account for irradiance lamp drift. The final assignment of spectral irradiance is simply attributed to the lamp as of the midpoint of the burning time. Between scale realizations when the group of four primary working standards is being used as a basis for calibrating additional lamps, their drifts are taken into account. Various empirical drift models have been used [3]. The present drift equation is

$$E_{\lambda }=A+B\cdot t,$$ (10)

where:

*t* is time in burning hours,
*A* and *B* are constants determined by fitting.

The fitting is performed independently at each wavelength.

Drift of the spectral radiance of the integrating sphere source is taken into account by simple linear interpolation in time between the initial and final spectral radiance values.

An interpolation equation was developed for calculating the spectral irradiance of tungsten halogen lamps at wavelengths between the 31 calibrated wavelengths. This equation is

$$E_{\lambda }=(A_{0}+A_{1}\cdot \lambda +\ldots +A_{n}\cdot \lambda^{n})\cdot \lambda^{-5}\cdot \exp(a+b/\lambda ).$$ (11)

Setting the polynomial equal to 1, multiplying both sides by λ^5^, and taking the log of both sides gives ln(*E*_λ_·λ^5^)=*a*+*b*/λ, in which it will be recognized that exp(*a*) is an effective gray-body emissivity and *b* is closely related to the reciprocal of the distribution temperature. A least squares fitting using a weighting of 1 is performed to determine *a* and *b*. With a and b thus fixed, eq (11) is least squares fitted using a weighting of $1/{E_{\lambda }}^{2}$ (assuming constant percentage measurement error) to determine *A*_0_, *A*_1_, … *A_n_*. In practice it has been found that the final fit is considerably improved if the spectrum is broken into two spectral regions, 250 to 400 nm and 350 to 1600 nm, for separate fitting. See reference [3] for examples of fitting eq (11) to lamp data. This method is only valid for the continuous spectrum and does not predict emission lines and absorption bands. Spectral irradiance values predicted using eq (11) have an uncertainty of about 0.5%.

## 8. Uncertainty Estimation

The spectral irradiance scale uncertainty analysis is broken down into three parts. First, the uncertainty in the spectral radiance of the integrating sphere source is determined. Second, the uncertainty in the transfer to the spectral irradiance primary working standards is determined. Third, the uncertainty in the transfer from the primary working standards to the irradiance test lamps is determined. The overall uncertainty in the primary working standards is determined by combining in quadrature the first and second parts. The overall uncertainty in a group of test lamps is determined by combining in quadrature all three parts. All uncertainties are estimated at the 3*σ* level.

### 8.1 Integrating Sphere Source Spectral Radiance Uncertainty

The uncertainties in the spectral radiance values assigned to the integrating sphere source are obtained from the observed precision of the measurements and the estimated systematic error in both the measured and the provided quantities (e.g., temperature of melting gold). Uncertainties obtained from the observed precision and from the published values of the physical constants are based upon three standard deviations. Uncertainties of systematic errors are estimated at the equivalent of three standard deviations.

In order to examine the contributions of the various errors to the uncertainty in the spectral radiance of the integrating sphere source, an approximate equation for the complete measurement process was derived by using the Wien approximation to the Planck relation. The details of the derivation are described in reference [6]. The resulting equation is

$$\begin{array}{l} L_{\lambda }≅(s_{\lambda }\cdot \epsilon_{B}\cdot d\cdot M_{\lambda })\left[c_{1}/[\pi \cdot \lambda^{5}\cdot (e^{c_{2}/\lambda \cdot T_{\text{Au}}})]\right]\\ \;\cdot {\left(s_{r}\cdot f_{r}\cdot M_{r}/\epsilon_{B}\right)}^{\lambda_{r}/\lambda }, \end{array}$$ (12)

where, with VTBB denoting the variable-temperature blackbody and GPBB denoting the gold-point blackbody, the definitions of the quantities are:

*M*_λ_, signal ratio of the VTBB-integrating sphere source comparison,
*M*_r_, signal ratio of the GPBB-VTBB comparison,
*s*_λ_, size-of-source correction for the VTBB-integrating sphere source comparison,
ϵ_B_, effective emissivity of the VTBB,
*d*, correction for integrating sphere source drift during calibration,
*s*_r_, size-of-source correction for the GPBB-VTBB comparison,
*f*_r_, linearity-range factor correction,
*T*_AU_, IPTS-68 temperature of melting gold,
*c*_1_, first radiation constant,
*c*_2_, second radiation constant,
λ, wavelength of the VTBB-integrating sphere source comparison,
λ_r_, wavelength of the GPBB-VTBB comparison, 654.6 nm.

Spectral radiance uncertainties due to the factors of eq (12) are obtained from the partial derivative with respect to those factors and the estimated uncertainty in the factor. Differences between errors calculated by eq (12) and those calculated by the exact Planck relation are negligible. Note that for the wavelengths λ and λ_r_ this process yields the error due to inserting the wrong wavelength in the spectral radiance calculation, not the error due to an incorrect wavelength setting.

In addition to the factors which appear explicitly in eq (12), uncertainties in the ratios *M*_λ_ and *M*_r_ arise from errors in the wavelength settings λ (0.1 nm) and λ_r_ (0.05 nm), in the current measurements of the vacuum pyrometer lamps (0.2 mA) and the integrating sphere source lamp (0.3 mA), and in the measured spectral responsivity function. The uncertainties in the ratios due to wavelength setting and electric current are assessed at a number of wavelengths by measurement of the change in signal ratio when varying these quantities. This technique for determining the effect upon the signal ratios due to the uncertainties in the measured spectral responsivity function is derived in reference [20]. The spectral radiance uncertainties due to these factors are then deduced from the ratio uncertainties as before. The signal ratio, lamp current, and wavelength setting errors are considered random; the remaining errors are systematic.

Table 1 lists the uncertainties obtained by this process. The calculated uncertainties, in percent of spectral radiance, are tabulated for a number of wavelengths over the calibration range. The individual values are combined in quadrature to yield the combined uncertainty for each wavelength. These uncertainties apply to-the spectral radiances values of the integrating sphere source.

### 8.2 Radiance to Irradiance Transfer Uncertainty

The uncertainty in the transfer from the integrating sphere source to the spectral irradiance primary working standards is obtained from examining the contributions of the various errors in the following measurement equation,

$$E_{\lambda }(\text{PWS})=m\cdot d_{1}\cdot f\cdot (S_{\text{PWS}}/S_{\text{ISS}})\cdot L_{\lambda }(\text{ISS})\cdot [(\pi \cdot {r_{1}}^{2})/R^{2}],$$ (13)

where:

*E*_λ_ (PWS), spectral irradiance of a primary working standard,
*m*, mapping correction for the average spectral radiance of the integrating sphere source,
*d*_1_, integrating sphere source drift correction,
*f*, linearity-range factor correction,
*S*_PWS_*/S*_ISS_, signal ratio of the primary working standard-integrating sphere source comparison,
*L*_λ_ (ISS), spectral radiance of the integrating sphere source,
(π·*r*_1_^2^)*/R*^2^, geometric factor in the irradiance calculation [see eqs (7), (8a), and (9)]

In addition to the factors which appear explicitly in eq (13), uncertainties in the ratio *S*_PWS_*/S*_ISS_, arise from errors in the wavelength settings and in the electrical current measurements of the sources. There are also uncertainties due to spectral scattering, stray light, and averaging sphere responsivity. All these uncertainties have been evaluated and are listed in table 2.

### 8.3 Test Lamp Irradiance Transfer Uncertainty

The uncertainty in the transfer from the spectral irradiance primary working standards to a group of irradiance test lamps is obtained from examining the contributions of the various errors in the following measurement equation,

$$E_{\lambda }(\text{TL})=f\cdot (S_{\text{TL}}/S_{\text{PWS}})\cdot E_{\lambda }(\text{PWS}),$$ (14)

where:

*E*_λ_ (TL), spectral irradiance of a test lamp,
*f*, linearity-range factor correction,
*S*_TL_/*S*_PWS_, signal ratio of the test lamp-primary working standard comparison,
*E*_λ_ (PWS), spectral irradiance of a primary working standard.

In addition to the factors which appear explicitly in eq (14), uncertainties in the ratio *S*_TL_*/S*_PWS_ arise from errors in the wavelength settings and in the electrical current measurements of the sources. All these uncertainties have been evaluated and are listed in table 3.

### 8.4 Overall Uncertainty of the Primary Working Standards

Table 4 lists the overall uncertainties of the primary working standards. It is made up by combining the results of tables 1 and 2. The differences between lines 1a and 1b (and between 3a and 3b) are caused by the systematic uncertainty introduced by an assumed uncertainty of 0.4 K in the gold-point temperature.

### 8.5 Overall Uncertainty of a Group of Test Lamps

Table 5 lists the overall uncertainties of a group of test lamps. It is made up by combining the results of tables 1, 2, and 3 and adding a model error. The model error is necessary because the primary working standards drift with time. A time drift model is applied for each of the primary working standards [see eq (10)] but the possibility that this drift may be wrong introduces an additional uncertainty in table 5, but not included in table 2 or table 4. This uncertainty was obtained by comparing the calculated extrapolated spectral irradiance with further scale realizations. When the primary working standards are used between scale realizations, this additional uncertainty must be combined in quadrature with the other uncertainties.

The differences between lines 1a and 1b (and between 4a and 4b) are again caused by the systematic uncertainty introduced by an assumed uncertainty of 0.4 K in the gold-point temperature.

## Figures and Tables

**Table 1:** Integrating sphere source spectral radiance uncertainty (3*σ*) in percent

	Wavelength (nm)							
Source of error	250	350	654.6	900	1300	1600	2000	2400
*T*_Au_ (s)	1.29	0.92	0.49	0.36	0.25	0.20	0.16	0.13
*M*_r_ (r)	0.16	0.11	0.08	0.20	0.17	0.12	0.09	0.36
*M*_λ_ (r)	0.25	0.18	0.08	0.20	0.22	0.33	0.66	1.08
*s*_r_ (s)	0.26	0.19	0.10	0.07	0.05	0.04	0.03	0.03
*s*_λ_ (s)	0.10	0.10	0.10	0.10	0.10	0.10	0.10	0.10
*f*_r_ (s)	0.26	0.19	0.10	0.07	0.05	0.04	0.03	0.03
*d* (s)	0.10	0.10	0.10	0.10	0.10	0.10	0.10	0.10
ϵ_B_ (s)	0.16	0.09	0.00	0.03	0.05	0.06	0.07	0.07
λ_r_ setting (r)	0.15	0.08	0.04	0.02	0.01	0.00	0.01	0.01
λ setting (r)	0.03	0.04	0.03	0.01	0.02	0.01	0.01	0.01
*c*_1_ (s)	0.00	0.00	0.00	0.00	0.00	0.00	0.00	0.00
*c*_2_ (s)	0.13	0.10	0.05	0.04	0.03	0.02	0.02	0.01
Lamp currents:								
Quinn-Lee (r)	0.11	0.08	0.04	0.03	0.02	0.02	0.01	0.01
1530 K (r)	0.05	0.04	0.02	0.02	0.01	0.01	0.01	0.01
ISS (r)	0.08	0.06	0.03	0.02	0.02	0.01	0.01	0.01
Spect. resp. (s)	0.08	0.06	0.03	0.02	0.02	0.02	0.01	0.01
Quadrature sum	1.41	1.01	0.55	0.49	0.41	0.44	0.70	1.16
Quadrature sum without *T*_Au_	0.58	0.42	0.25	0.34	0.33	0.39	0.69	1.15

Notes: Random errors denoted by (r), systematic errors by (s).

Sources of error described in section 8.1.

**Table 2:** Radiance to irradiance transfer uncertainty (3*σ*) in percent

	Wavelength (nm)							
Source of error	250	350	654.6	900	1300	1600	2000	2400
*s*_PWS_*/S*_ISS_ (r)	0.42	0.08	0.06	0.84	0.86	1.46	2.60	5.73
*f* (s)	0.26	0.19	0.10	0.07	0.05	0.04	0.03	0.03
*m* (s)	0.10	0.10	0.10	0.10	0.10	0.10	0.10	0.10
*d*_1_ (s)	0.10	0.10	0.10	0.10	0.10	0.10	0.10	0.10
λ (r)	0.02	0.02	0.02	0.02	0.02	0.02	0.02	0.02
Lamp currents:								
ISS (r)	0.08	0.06	0.03	0.02	0.02	0.01	0.01	0.01
PWS (r)	0.08	0.06	0.03	0.02	0.02	0.01	0.01	0.01
Geom. Factor (s)	0.20	0.20	0.20	0.20	0.20	0.20	0.20	0.20
Spec. Scat. (s)	0.05	0.05	0.05	0.05	0.05	0.05	0.05	0.05
Stray Light (s)	0.02	0.02	0.02	0.02	0.02	0.02	0.02	0.02
Av. Sph. Resp. (s)	0.01	0.01	0.01	0.01	0.01	0.01	0.01	0.01
Systematic error	0.36	0.31	0.27	0.26	0.26	0.25	0.25	0.25
Random error	0.43	0.11	0.08	0.84	0.86	1.46	2.60	5.73
Quadrature sum	0.57	0.33	0.28	0.88	0.90	1.48	2.61	5.74

Notes: Random errors denoted by (r), systematic errors by (s).

Sources of error described in section 8.2.

**Table 3:** Test lamp irradiance transfer uncertainty (3*σ*) in percent

	Wavelength (nm)							
Source of error	250	350	654.6	900	1300	1600	2000	2400
*s*_TL_/*S*_PWS_ (r)	0.87	0.21	0.15	0.42	0.68	0.72	1.59	2.60
*f* (s)	0.01	0.01	0.01	0.01	0.01	0.01	0.01	0.01
Lamp currents:								
PWS (r)	0.08	0.06	0.03	0.02	0.02	0.01	0.01	0.01
TL (r)	0.08	0.06	0.03	0.02	0.02	0.01	0.01	0.01
Systematic error	0.01	0.01	0.01	0.01	0.01	0.01	0.01	0.01
Random error	0.88	0.22	0.16	0.42	0.68	0.72	1.59	2.60
Quadrature sum	0.88	0.22	0.16	0.42	0.68	0.72	1.59	2.60

Notes: Random errors denoted by (r), systematic errors by (s).

Sources of error described in section 8.3.

**Table 4:** 1986 spectral irradiance scale uncertainty (3*σ*) in percent (derived from tables 1 and 2)

	250 nm	350 nm	654.6 nm	900 nm	1300 nm	1600 nm	2000 nm	2400 nm
1. NBS spectral radiance scale								
a. Absolute error (with respect to SI units)	1.41	1.01	0.55	0.49	0.41	0.44	0.70	1.16
b. NBS long term reproducibility (without *T*_AU_, see table 1)	0.58	0.42	0.25	0.34	0.33	0.39	0.69	1.15
2. Radiance to irradiance transfer								
a. Systematic errors	0.36	0.31	0.27	0.26	0.26	0.25	0.25	0.25
b. Random errors (3 *σ* precision)	0.43	0.11	0.08	0.84	0.86	1.46	2.60	5.73
3. Spectral irradiance scale uncertainty (quadrature sum)								
a. With respect to SI units	1.52	1.06	0.62	1.01	0.99	1.55	2.71	5.85
b. NBS long term reproducibility	0.81	0.53	0.38	0.94	0.96	1.53	2.70	5.85

**Table 5:** 1986 spectral irradiance scale transfer uncertainty (3*σ*) in percent (derived from tables 1, 2, and 3)

	250 nm	350 nm	654.6 nm	900 nm	1300 nm	1600 nm	2000 nm	2400 nm
1. NBS spectral radiance scale								
a. Absolute error (with respect to SI units)	1.41	1.01	0.55	0.49	0.41	0.44	0.70	1.16
b. NBS long term reproducibility	0.58	0.42	0.25	0.34	0.33	0.39	0.69	1.15
2. Radiance to irradiance transfer								
a. Systematic errors	0.36	0.31	0.27	0.26	0.26	0.25	0.25	0.25
b. Random errors (3*σ*, precision)	0.43	0.11	0.08	0.84	0.86	1.46	2.60	5.73
c. Model error	1.38	0.80	0.78	0.77	0.77	0.82	1.00	1.20
3. Test lamp irradiance transfer								
a. Systematic errors	0.01	0.01	0.01	0.01	0.01	0.01	0.01	0.01
b. Random errors (3*σ* precision)	0.88	0.22	0.16	0.42	0.68	0.72	1.59	2.60
4. Uncertainty of reported values (quadrature sum)								
a. With respect to SI units	2.23	1.35	1.01	1.34	1.42	1.89	3.29	6.51
b. NBS long term reproducibility	1.83	0.99	0.88	1.29	1.40	1.88	3.29	6.51
